# Functional Polymorphisms of *CHRNA3* Predict Risks of Chronic Obstructive Pulmonary Disease and Lung Cancer in Chinese

**DOI:** 10.1371/journal.pone.0046071

**Published:** 2012-10-03

**Authors:** Lei Yang, Fuman Qiu, Xiaoxiao Lu, Dongsheng Huang, Guanpei Ma, Yuan Guo, Min Hu, Yumin Zhou, Mingan Pan, Yigang Tan, Haibo Zhong, Weidong Ji, Qingyi Wei, Pixin Ran, Nanshan Zhong, Yifeng Zhou, Jiachun Lu

**Affiliations:** 1 School of Public Health, The Institute for Chemical Carcinogenesis, The State Key Lab of Respiratory Disease, Guangzhou Medical University, Guangzhou, Guangdong, China; 2 Guangzhou Institute of Respiratory Diseases, The First Affiliated Hospital, The State Key Lab of Respiratory Disease, Guangzhou Medical University, Guangzhou, Guangdong, China; 3 Department of Respiratory Medicine, Guangzhou Chest Hospital, Guangzhou, Guangdong, China; 4 The third Affiliated Hospital of Guangzhou Medical University, Guangzhou, Guangdong, China; 5 Department of Respiratory Medicine, the third Affiliated Hospital of Sun Yat-sen University, Guangzhou, Guangdong, China; 6 Department of Respiratory Medicine, Guangzhou Red Cross Hospital, Guangzhou, Guangdong, China; 7 Soochow University Laboratory of Cancer Molecular Genetics, Medical College of Soochow University, Suzhou, China; 8 Department of Epidemiology, The University of Texas M.D. Anderson Cancer Center, Houston, United States of America; Emory University, United States of America

## Abstract

Recently, several genome-wide association studies (GWAS) have identified many susceptible single nucleotide polymorphisms (SNPs) for chronic obstructive pulmonary disease (COPD) and lung cancer which are two closely related diseases. Among those SNPs, some of them are shared by both the diseases, reflecting there is possible genetic similarity between the diseases. Here we tested the hypothesis that whether those shared SNPs are common predictor for risks or prognosis of COPD and lung cancer. Two SNPs (rs6495309 and rs1051730) located in nicotinic acetylcholine receptor alpha 3 (*CHRNA3)* gene were genotyped in 1511 patients with COPD, 1559 lung cancer cases and 1677 controls in southern and eastern Chinese populations. We found that the rs6495309CC and rs6495309CT/CC variant genotypes were associated with increased risks of COPD (OR = 1.32, 95% C.I. = 1.14–1.54) and lung cancer (OR = 1.57; 95% CI = 1.31–1.87), respectively. The rs6495309CC genotype contributed to more rapid decline of annual Forced expiratory volume in one second (FEV1) in both COPD cases and controls (*P*<0.05), and it was associated with advanced stages of COPD (*P* = 0.033); the rs6495309CT/CC genotypes conferred a poor survival for lung cancer (HR = 1.41, 95%CI = 1.13–1.75). The luciferase assays further showed that nicotine and other tobacco chemicals had diverse effects on the luciferase activity of the rs6495309C or T alleles. However, none of these effects were found for another SNP, rs1051730G>A. The data show a statistical association and suggest biological plausibility that the rs6495309T>C polymorphism contributed to increased risks and poor prognosis of both COPD and lung cancer.

## Introduction

COPD and lung cancer are the most strikingly increasing lung diseases with ranks of the fourth cause of death and the first cancer-related death worldwide, respectively [1], [2]. Smoking is the major risk factor for both diseases, about 20–30% of smokers develop COPD and 10–15% of smokers develop lung cancer [3], [4]. Several common pathological mechanisms involving in both diseases have been proposed, especially the long-term inflammatory process [5], [6] and the epithelial- mesenchymal transition (EMT), which are thought to cause lung carcinogenesis during the COPD period [7], reflecting COPD a risk factor of lung cancer [8]. Because both COPD and lung cancer are inheritable, the genetic characteristics conferring this dual susceptibility might overlap [9].

Recently, eleven genome-wide association studies (GWAS) have reported several susceptibility loci for COPD and lung cancer [10]–[20]. Among them, five studies were performed for COPD in whites like Norwegian, European Americans, non-Hispanic Americans [10]–[14] and six studies were performed for lung cancer in various populations including Japanese, Korean, Chinese, Americans of European ancestry and British [15]–[20]. Remarkably, all these studies have revealed that the single nucleotide polymorphisms (SNPs) were located in nicotinic acetylcholine receptor genes (*CHRNA3*, *CHRNB4*, *CHRNA5*) which were mapped to chromosome 15 q25 are shared by the two diseases [10], [11], [14]–[18]. For example, an GWAS conduced in Norwegian by using Illumina's HumanHap550 genotyping Bead Chip reported that the SNP rs1051730 of *CHRNA3* was significantly associated with COPD risk (*P* = 5.74**×**10^−10^) [11], and another study conducted in American reported that the SNP rs8042374 in 15 q25 was susceptibility loci for lung cancer (*P* = 7.75**×**10^−12^) [16]. However, this shared genetic etiology may simply be due to nicotine- dependence because these SNPs are also associated with smoking behavior [21], [22]. Yet, controversial findings were reported in never smokers. Studies in American and British populations showed that these SNPs were not associated with risk of lung cancer in never smokers, but studies in some other European, Japanese and Chinese populations reported a significant association in never smokers [17], [23]–[25]. It is likely that because there were passive smokers included as never-smokers in these studies, such misclassification may have biased the associations.

Genetic effects of some susceptible SNPs may differ between different ethnics. A recent Chinese study reported that the two most significant causal SNPs (i.e., rs1051730, rs8034191) shared by lung cancer and COPD in European populations were not associated with lung cancer risk in Chinese population [25]. They found a functional SNP (rs6495309) in the *CHRNA3* gene that exerted an effect on regulating gene expression, leading to an increased lung cancer risk. Intriguingly, this SNP was also associated with COPD risk in Norwegians [11]. However, no study has investigated these SNPs in COPD in Chinese population.

COPD and lung cancer are closely related, to simultaneously study these susceptible SNPs in both diseases would reveal the genetic mechanisms shared by these diseases, which may explain why lung cancer incidence is high in COPD patients. Furthermore, smoking is always associated with poor prognosis of COPD and lung cancer [26], [27], these SNPs of nicotine-related genes may have some effects on prognosis of COPD or lung cancer patients. Therefore, in current study, we investigated the associations between two SNPs (rs1051730 and rs6495309) in the *CHRNA3* gene and risk as well as prognosis of COPD and lung cancer in southern and eastern Chinese populations. We further analyzed the functionality of these polymorphisms with biological assays.

## Methods

### Study Subjects

We conducted two hospital-based case-control studies in southern and eastern Chinese populations. Briefly, the southern Chinese population included 1025 COPD patients, 1056 lung cancer patients, and 1061 normal controls was used as a discovery set and the eastern Chinese population included 486 COPD patients, 503 lung cancer cases and 616 controls was used as a validation set. The lung cancer patients have been described previously [28]–[31];Definition of COPD was according to the global initiative for chronic obstructive lung disease [2], the controls were normal lung function and were age (±5 years) and sex frequency-matched with COPD cases. Furthermore, there were 510 lung cancer patients from southern China and 296 cases from eastern China who had complete survival outcomes data of death directly caused by lung cancer or survivorship, and 116 COPD patients and 357 controls had at least four years follow-up between 2002 and 2010 with annual spirometric detections [26], [32], [33]. All the participants were ethnic Han Chinese and they shared no kinship with each other, and none had blood transfusion in the last 6 months. The participant was asked to provide data on smoking status, pre-existing COPD and other factors and to donate 5 ml peripheral blood after an informed consent was obtained from all participants in written form. Additional detail on the samples recruitment and the definition of smoking status and other factors were provided **in Appendix S1** and elsewhere [28]–[30], [34]. The studies were approved by the institutional review boards of Guangzhou Medical University (Ethics Committee of Guangzhou medical university: GZMC2007-07-0676) and Soochow University (Ethics Committee of Soochow University: SZUM2008031233).

### SNP Selection and Genotyping Analysis

Based on the data of published GWAS in COPD or lung cancer, we set up a database containing reported SNPs and *P* values of each association for the diseases, and we searched the shared loci by both diseases with a self-made excel file (**File S1**). Seven SNPs were identified (i.e., rs1051730, rs1394371, rs1996371, rs4887077, rs6495309, rs667282 and rs8034191; **Figure S1**). All these SNPs were located in chromosome 15 q25, and five SNPs (rs1051730, rs1394371, rs1996371, rs4887077 and rs8034191) were in highly linkage disequilibrium (LD) with each other (D’ = 1.00, r^2^>0.80), and the other two SNPs rs6495309, rs667282 were also in highly LD (D’ = 0.90, r^2^ = 0.71) (**Figure S2**). Therefore, we selected two SNPs (rs1051730G>A: Y215Y; rs6495309T>C: −2109 bp to the transcription initial site ATG) as representative SNPs that cover the genetic information of above all reported GWAS SNPs.

SNP genotyping was performed by the PCR-RFLP method. Briefly, primers [5′- GCC ATC ATC AAA GCC CCA GGC TT-3′ (forward) and 5′- GGC AGG TAG AAG ACG AGC AC-3′ (reverse)] and the enzyme *Dra*I (New England BioLabs, Ipswich, MA, USA) were used to identify the rs1051730G>A genotypes. Primers [5′- CTC CTG GCA TTC AGC AAA-3′ (forward) and 5′- AGG CGG CAG ATC ACC TAA-3′ (reverse)] and the enzyme *Nla*III (New England BioLabs) were used to identify the rs6495309T>C genotypes. 10% samples were randomly selected to perform repeated assays for each SNP, and the results were 100% concordant. We also randomly selected 100 samples for direct sequencing to confirm the genotyping results, and the results were also 100% concordant (**Figure S3**).

### Luciferase Assays

Two luciferase reporter plasmids contained the *CHRNA3* promoter region with either rs6495309T or C allele were gifted from Dr. Cheng Wu and Zhibin Hu [25]. We detected the SNPs' effects on the promoter activity in the host cells under the treatment of tobacco extract or tobacco chemical carcinogens [i.e., nicotine, Nicotine-derived nitrosamine ketone (NNK) or Benzo[a]pyrene (B(a)P)]. Tobacco extract was self prepared as described by Nakamura et al. [35]. Briefly, the smog of two lit cigarettes was collected by a syringe-pump and was sent to 50 ml DMEM-F12. After sealing and complete mixing, 1 M NaOH was used to adjust its pH to 7.4 and filter membrane (pore size: 0.22 µm) was used to remove bacteria. We seeded 5×10^5^ human lung cell lines including 16HBE (an immortal human bronchial epithelial cell line), A549 (a human lung adenocarcinoma cell line) and H460 (a human lung large cell carcinoma cell line) into 24-well plates and transfected them with a pGL3-basic construct with rs6495309C or T allele. The three cell lines were purchased from Cell Bank of Type Culture Collection of Chinese Academy of Sciences, Shanghai Institute of Cell Biology, Chinese Academy of Sciences. pRL-TK plasmid (Promega) was co-transfected as a normalized control. All transfections were carried out in triplicate. After the cells were cultured for 14 hour, a final concentration of 10 uM nicotine, 100 nM NNK or 1 uM B(a)P were added into the cultures, respectively [36]. The activities of the *CHRNA3-*pGL3 reporter with firefly luciferase and the internal standard reporter with Renilla luciferase were quantified by a Dual-Luciferase Reporter Assay System (Promega, Madison, WI, USA) after 1 hour treatment.

### Statistical Analysis

The Chi-square test was used to compare among groups of categorical variables between cases and controls as well as Hardy-Weinberg equilibrium test in controls. Associations between the SNPs and COPD as well as lung cancer risks were estimated using an unconditional logistic regression model with adjustment for age, sex, smoking status, and drinking status. The best genetic-effect model for each SNP on diseases risk was estimated based on the smallest Akaike’s information criterion (AIC) value [37]. 10,000 permutation tests were used to estimate exact *P* values of the associated *P* value for COPD or lung cancer risk. Stratification analysis was performed to show the effect of possible confounder factors on the association between CHRNA3 genotypes and lung diseases risk, and gene-environment interactions on COPD or lung cancer risk as well as lung cancer survival were analyzed with a multiplicative interaction as when OR 11> OR 10 × OR 01, in which OR 11 = the OR when both factors were present, OR 01 = the OR when only factor 1 was present, OR 10 = the OR when only factor 2 was present [38]. Breslow-Day Test was used to analyze the homogeneity between stratum-ORs in stratification analysis. The power and sample size Calculation (PS) software (http://biostat.mc.vanderbilt.edu/twiki/bin/view/Main/PowerSampleSize) was used to calculate the statistical power. One-way ANOVA test and the linear regression model with adjustment for age, sex, smoking status, and drinking status were used for analyzing the annual decline of pre-bronchodilator FEV1 by *CHRNA3* genotypes and surrounding factors such as age, sex, smoking status, pack-year smoked, drinking status, cooking with coal, biomass using, and COPD stages. The Kaplan-Meier method, log-rank test, and Cox proportional hazards regression model with adjustment for age, sex, smoking status, histology, stages, surgery, chemotherapy, and radiotherapy were used to evaluate the effects of *CHRNA3* genotypes as well as the above surrounding factors and clinical treatments on overall survival of lung cancer patients. The difference of luciferase activity was analyzed by Student’s *t* test. A two-tailed *P*<0.05 was considered statistically significant, and the SAS software (version 9.1; SAS Institute, Cary, NC, USA) was used for all analyses.

## Results

### CHRNA3 Genotypes and Risks of COPD or Lung Cancer

Demographics, selected variables and clinical information of COPD and lung cancer patients as well as healthy controls are presented in **Table S1.** As shown, smoking status, pack-year smoked, cooking with coal, biomass using are common risk factors for both the diseases (*P*<0.05 for all).

The frequency distributions of genotypes of the two SNPs in individuals were presented in **Table 1**. In the discovery set, we found significant associations between rs6495309T>C genotypes as well as alleles and both diseases risks (*P*<0.05 for all). According to the smallest AIC value, the rs6495309CC genotype exerted a significantly increased risk of COPD under recessive genetic model (OR = 1.33; 95% CI = 1.10–1.60; *P* = 0.003), while the rs6495309CT/CC genotypes were associated with lung cancer risk under dominant genetic model (OR = 1.51; 95% CI = 1.21–1.87; *P* = 2.2×10^−4^). However, no significant association between both diseases and the genotypes nor alleles were found for the SNP rs1051730G>A (*P*>0.05 for all).

**Table 1:** Distribution of genotypes in *CHRNA3* gene and associations with risk of COPD and lung cancer.

	Discovery Set (Southern Chinese)					Validation Set (Eastern Chinese)				
Genotypes/Alleles	Controlsn (%)^b^	COPDn (%)	AdjustedOR(95% CI)^a^	Lungcancern (%)	Adjusted OR(95% CI) a	Controlsn (%)^b^	COPDn (%)	Adjusted OR(95% CI)^a^	Lungcancern (%)	AdjustedOR(95% CI)^a^
Total no. ofsubjects	1061	1025		1056		616	486		503	
Total no. ofalleles	2122	2050		2112		1232	972		1006	
**rs6495309T>C**										
TT	250(23.6)	227(22.1)	1.00 (ref.)	181(17.1)	1.00 (ref.)	148(24.0)	114(23.4)	1.00 (ref.)	81(16.1)	1.00 (ref.)
CT	502(47.3)	433(42.2)	0.94(0.75–1.17)	497(47.1)	**1.38(1.10–1.74)**	292(47.4)	204(42.0)	0.89(0.66–1.21)	238(47.3)	**1.46(1.05–2.77)**
CC	309(29.1)	365(35.7)	**1.27(1.01–1.61)**	378(35.8)	**1.71(1.33–2.19)**	176(28.6)	168(34.6)	1.23(0.88–1.70)	184(36.6)	**1.95(1.37–2.77)**
Group test*P* value			**0.006**		**1.24×10** ^−**4**^		0.084		**0.001**	
C allele	0.528	0.567		0.593		0.523	0.556		0.602	
Allelic test*P* value			**0.009**		**1.77×10** ^−**5**^			0.125	**1.60×10** ^−**4**^	
Additive model			**1.14(1.01–1.28)**		**1.30(1.15–1.47)**			1.10(0.94–1.30)		**1.37(1.15–1.63)**
Dominant model										
TT	250(23.6)	227(22.1)	1.00 (ref.)	181(17.1)	1.00 (ref.)	148(24.0)	114(23.4)	1.00 (ref.)	81(16.1)	1.00 (ref.)
CT+CC	811(76.4)	798(77.9)	1.07(0.88–1.31)	875(82.9)	**1.51(1.21–1.87)**	468(76.0)	372(76.6)	1.01(0.77–1.35)	422(83.9)	**1.66(1.21–2.26)**
Recessive model										
TT+CT	752(70.9)	660(64.4)	1.00 (ref.)	678(64.2)	1.00 (ref.)	440(71.4)	318(65.4)	1.00 (ref.)	319(63.4)	1.00 (ref.)
CC	309(29.1)	365(35.6)	**1.33(1.10–1.60)**	378(35.8)	**1.36(1.13–1.64)**	176(28.6)	**168(34.6)**	**1.32(1.02–1.71)**	184(36.6)	**1.49(1.15–1.93)**
**rs1051730G>A**										
GG	1025(96.6)	988(96.2)	1.00 (ref.)	1007(95.4)	1.00 (ref.)					
GA	36(3.4)	39(3.8)	1.10(0.67–1.84)	49(4.6)	1.33(0.79–2.14)					
Group test*P* value										
A allele	0.017	0.019		0.023						
Allelic test*P* value			0.623		0.148					

a Adjusted in a logistic regression model that included age, sex, smoking status and drinking status.

b The observed genotype frequencies among the control subjects were all concordant with the Hardy-Weinberg equilibrium in the two independent populations (*P*>0.05).

We only validated the associations of rs6495309T>C in eastern Chinese and the results were consistent. The carriers of rs6495309CC genotype had a 1.32-fold increased COPD risk (OR = 1.32, 95% C.I. = 1.02–1.71; *P* = 0.036) and the rs6495309CT/CC genotypes had a 1.66-fold increased lung cancer risk (OR = 1.66, 95% C.I. = 1.21–2.26; *P* = 0.002). The associations were homogeneous in two datasets (COPD, *P* = 0.907; lung cancer, *P* = 0.549), the rs6495309CC and rs6495309CT/CC genotypes conferred to increased risks of COPD (OR = 1.32, 95% C.I. = 1.14–1.54, *P* = 1.3×10^−4^) and lung cancer (OR = 1.57; 95% CI = 1.31–1.87, *P* = 2.2×10^−7^), respectively (**Figure S4**). Meanwhile, the permutation test further confirmed above associations after correction for 10,000 testing resampling (corrected *P* = 0.001 for COPD; *P* = 5.9×10^−6^ for lung cancer, respectively).

COPD contributes to an increased risk of lung cancer, thus the adverse genotypes may have an additive effect on increasing lung cancer risk with pre-existing COPD. In those subjects with pre-existing COPD, rs6495309C genotypes had an intuitively higher risk of lung cancer (OR = 1.83, 95% CI = 1.04–3.21, *P* = 0.024) compared to those subjects without pre-existing COPD (OR = 1.50, 95% CI = 1.24–1.81, *P* = 1.47×10^−5^), but these ORs were not statistically different (Breslow-Day test: *P* = 0.364, data not shown).

### Stratification Analysis

We merged the two populations in stratification analysis to increase the study power. Only data for the rs6495309T>C polymorphism were presented, because the rs1051730G>A had no further positive findings. As shown in **Figure S4**, there were significant differences of the associations between the *CHRNA3* genotypes and the increased risk of COPD as well as lung cancer in smoking status (Breslow-Day test: *P* = 0.043 for COPD; *P* = 0.002 for lung cancer) as the associations were all significant in smokers (*P*<0.05 for all), but not in never smokers (*P*>0.05 for all); and they were also significant for lung cancer risk (*P* = 0.028) and for COPD risk with a borderline significant (*P* = 0.098) in passive smokers. In addition, there was a significantly higher lung cancer risk in those with rs6495309CT/CC genotypes and passive smoking from parents (OR = 3.53; 95% CI = 1.73–7.20; *P* = 0.001) compared to those with non passive smoking from parents (Breslow-Day test: all *P* = 0.008) However, there was no significant difference in the effect of rs6495309T>C by age, sex, drinking status, cooking with coal, biomass using or clinical stages in both diseases.

**Figure 1:**
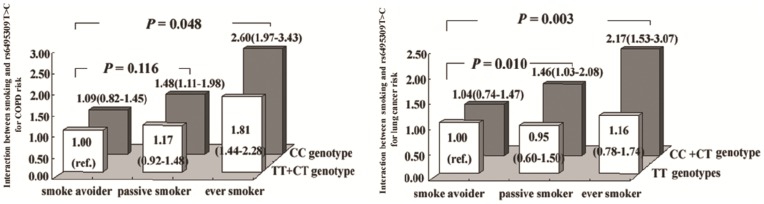
Interaction analysis between the rs6495309T>C polymorphism and smoking status on COPD or lung cancer risk. The smoker avoiders with rs6495309TT genotype are defined as reference. The rs6495309C variant genotype(s) interacted with smoking on risks of both diseases but only interacted with passive smoking on lung cancer risk.

Because an intuitively significant interaction was observed between the *CHRNA3* adverse genotypes and lung cancer risk (**Figure S4)**, we further showed interactions between the SNP rs6495309T>C and smoking status with three conditions (ever smoker, passive smoker, smoking avoider) on both COPD and lung cancer risks in **Figure 1**. As shown, significant interactions between ever smoking and rs6495309C genotypes were observed for risk of both diseases (*P = *0.048 for COPD; *P = *0.003 for lung cancer), and between passive smoking and rs6495309C genotypes on increasing lung cancer risk (*P = *0.010). In addition, as shown in **Figure S4,** passive smoking from parents also interacted with rs6495309CT/CC genotypes on increasing lung cancer risk (*P* = 0.005) and COPD risk with borderline significant (*P* = 0.051).

### CHRNA3 Genotypes and Pulmonary Function

**Figure 2:**
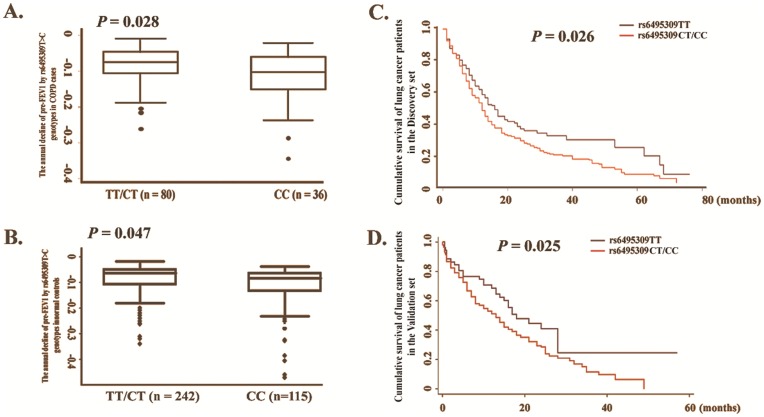
Prognosis analysis by the rs6495309T>C genotypes. **A,** The annual average decline of pre-bronchodilator FEV1 by rs6495309T>C genotypes in COPD patients. **B,** The annual average decline of pre-bronchodilator FEV1 by rs6495309T>C genotypes in healthy controls. **C,** Kaplan-Meier survival curve for lung cancer patients by rs6495309T>C genotypes in the Discovery set. **D,** Kaplan-Meier survival curve for lung cancer patients by rs6495309T>C genotypes in the validation set. The rs6495309CC variant genotype was associated with more rapid decline of pre-bronchodilator FEV1 in both COPD cases and controls, and the rs6495309C genotypes conferred a poor survival for lung cancer.

As shown in **Figure 2**, those rs6495309CC genotype carriers had a significant higher annual average decline of pre-FEV1 than that of CT/CC genotypes in 116 COPD patients (CC: n = 36, −0.115±0.086 L, CT/TT: n = 80, −0.080±0.059 L, Student’s *t* test: *P = *0.028, linear regression test *P* = 0.023; **Figure 2A**). Similar result was also observed in 357 control subjects (CC: n = 115, −0.076±0.077 L, CT/TT: n = 242, −0.095±0.098 L, Student’s *t* test: *P* = 0.047, linear regression test *P* = 0.045; **Figure 2B**). Furthermore, the rs6495309T>C polymorphism was significantly correlated with COPD progression exhibited by COPD Gold stages (*P* = 0.033).

In addition, smoking status and pack year smoked were associated with the annual decline of pre-bronchodilator FEV1 in both COPD cases and controls (*P*<0.05), while biomass using, COPD stages had a same effect in cases and sex had same effects in controls (*P = *0.032, **Table S2**).

### CHRNA3 Genotypes and Survival of Lung Cancer Patients

The survival analysis showed that age, smoking status, clinical stages, surgical operation, chemotherapy, and radio radiotherapy were all significantly associated with survival outcomes of lung cancer patients (*P*<0.05 for all; **Table S3**).

As shown in **Table 2**, a worse survival with a median survival time (MST) of 12 months was observed in the rs6495309CT/CC genotypes carriers than that of 15 months in rs6495309TT genotype carriers in southern Chinese (*P* = 0.026, **Figure 2C**). The Cox model analysis showed that the hazards ratio (HR) of rs6495309C genotypes on cancer death was statistically significant (HR = 1.34, 95% CI = 1.03–1.74, *P* = 0.028). The results in eastern Chinese further confirmed these findings, in which rs6495309 CT/CC genotypes carriers had a much lower MST (13 months) than rs6495309TT genotype carriers with an 18 MST (Log-rank test: *P* = 0.025; COX model: HR = 1.55, 95% C.I. = 1.04–2.30, *P* = 0.028; **Figure 2D**). The pooled analysis of two populations showed that the rs6495309CT/CC genotypes conferred a 1.41-fold death risk (95%CI = 1.13–1.75, *P* = 0.002) than rs6495309TT genotype for lung cancer patients.

**Table 2:** Analysis of *CHRNA3* rs6495309T>C and lung cancer survival.

rs6495309T>C	n (%)	Death	MST (months)	Log-rank *P* value	HR (95%CI)	COX mode *P* value a
**Discovery set**	510	413	12			
TT	94(18.4)	68	15	**0.069**	1.00(ref.)	
CT	241(47.3)	207	12		**1.38(1.05–1.82)**	**0.021**
CC	175(34.3)	138	12		1.13(0.98–1.31)	0.098
Trend test *P* value a					**0.172**	
TT	94(18.4)	68	15	**0.026**	1.00(ref.)	
CT+CC	416 (81.6)	345	12		**1.34(1.03–1.74)**	**0.028**
**Validation set**	296	208	12			
TT	51 (17.2)	29	18	0.079	1.00(ref.)	
CT	136 (45.9)	94	13		1.40(0.91–2.15)	0.127
CC	109 (36.8)	81	13		1.21(0.97–1.49)	0.085
Trend test *P* value a					0.077	
TT	51(17.2)	29	18	**0.025**	1.00(ref.)	
CT+CC	245 (82.8)	175	13		**1.55(1.04–2.30)**	**0.028**
**Merged Set**	806	617	12			
TT	145(18.0)	97	17	**0.005**	1.00(ref.)	
CT	486(60.3)	382	12		**1.46(1.17–1.82)**	**0.001**
CC	175(21.7)	138	12		**1.14(1.01–1.30)**	**0.044**
Trend test *P* value a					**0.073**	
TT	145(18.0)	680	17	**0.002**	1.00(ref.)	
CT+CC	661(81.0)	144	12		**1.41(1.13–1.75)**	**0.002**

Abbreviations: MST, median survival time; HR, hazard ratio;

a Cox regression analysis was adjusted for age, sex, smoking status, histology, stage, surgery, chemotherapy, and radiotherapy status.

The adverse genetic effect on lung cancer survival was then investigated in stratification analysis. As shown in **Figure S5**, the association between rs6495309CT/CC genotypes and poor lung cancer survival was more evident in male, in smokers, in subgroups of more pack-year smoked, in patients with clinical stage I or II, and in patients with squamous cell carcinoma type. Furthermore, there was no significant interaction between rs6495309T>C genotypes and the environmental factors on lung cancer survival (*P*>0.05 for all).

### Reporter Gene’s Activity

**Figure 3:**
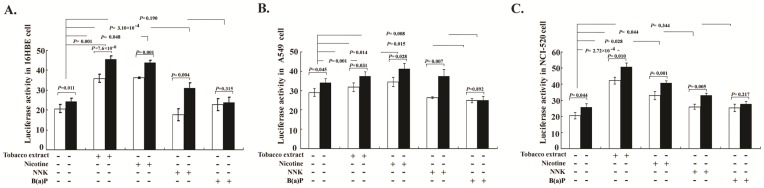
Luciferase expressions in different cell lines under the treatment of tobacco components. **A,** 16HBE. **B,** A549. **C,** NCI-520. Columns, mean from three independent experiments; bars, SD; and the difference of luciferase activity between C/T allele and the luciferase activity levels by chemical treatment (luciferase activity with chemical treatment – luciferase activity without treatment) were analyzed by Student’s *t* test Tobacco extract, nicotine and NNK could induce a significantly higher increased luciferase activity driven by the rs6495309C allele than that driven by the T allele.

As shown in **Figure 3**, we confirmed the higher reporter gene activity driven by the rs6495309C allele compared to that driven by the rs6495309T allele [25], and tobacco extract as well as nicotine could induce a higher promoter activity for both reporter genes with the rs6495309T or C allele (*P*<0.05 for all cell lines). Meanwhile, tobacco extract and nicotine could induce a significantly higher increased luciferase activity driven by the rs6495309C allele than that driven by the T allele (Student’s *t* test: *P*<0.05 for all). Moreover, we also found that NNK could also moderately increase the transcription activity of the rs6495309C construct than that of the rs6495309T construct (*P*<0.05 for all). However, no such effect was observed under the treatment of B(a)P.

## Discussion

Our current study revealed that the SNP rs6495309T>C in the *CHRNA3* gene was associated with an increased risks and poor prognosis of both COPD and lung caner in Chinese populations. The rs6495309C variant genotypes (CC or CT/CC) interacted with smoking on increasing risks of both diseases, and potentially with passive smoking on risk of lung cancer and COPD. The reporter assays showed that nicotine and NNK, but not B(a)P could induce a higher promoter activity of the rs6495309C allele than T allele. However, for the SNP rs1051730G>A, no significant association was observed for neither COPD nor lung cancer. To best of our knowledge, this is the first study to reveal the SNP in the *CHRNA3* gene was associated with both COPD and lung cancer risks and prognosis.

The biological function of the rs6495309C variant genotypes on increasing lung cancer risk had been described previously [25]. Here, we showed that the rs6495309T>C variation contributed to a reduction in the spirometric phenotypes. The rapid decline of FEV1 is a marker for airflow obstruction and used to assess the severity of the airflow obstruction [39], this is consistent with our finding that the rs6495309CC genotype was correlated with worse COPD stages. Studies have shown that CHRNA3 associated with smoking addiction through the high expression of CHRNA3 in the key regions of the brain [40], the rs6495309CC genotype carriers may consume more cigarettes, thus leading to more damage in pulmonary function [26], [41]. Consistently, lung cancer patients with the rs6495309C genotypes had poor survival, because smoking is always associated with a decreased survival of lung cancer [27], and CHRNA3 functions to promote chemotherapy resistance through the Akt-dependent proliferation and the NF-kappaB-dependent survival pathways under the stimulation of NNK [36], [42]–[44]. Taken together, we supported that the rs6495309T>C polymorphism is a available indicator of prognosis of both COPD and lung cancer.

By experimentally exposing the transfected cells with the plasmids to the tobacco related chemical components, we found that nicotine induced a higher level of promoter activity of *CHRNA3* under the control of the promoter with the rs6495309C allele than that with the T allele. Therefore, those rs6495309C allele carriers are more susceptible to be nicotine- dependence. Intriguingly, we also found that NNK conferred an increased transcript activity particularly for the *CHRNA3* promoter with the rs6495309C allele. Because *CHRNA3* is also a receptor of NNK, we could assume that NNK modulates the adverse genetic effect of rs6495309C genotypes on increasing lung diseases risk because NNK can cause gene mutation, DNA damage, activation of oncogenes and tumor-related signal pathways [45], [46].

In stratification analysis, the associations between the rs6495309C variant genotypes and COPD as well as lung cancer risk were all significant in smokers but not in never smokers; they were also significant for lung cancer risk, and borderline significant for COPD risk in passive smokers, which was novel findings. The CHRNA3 SNPs are associated with smoking behavior [21], [22]; it’s conceivable that the rs6495309C variant genotypes interacted with ever smoking on increasing COPD and lung cancer risk, and between the variant genotypes and passive smoking on increasing lung cancer risk, because of the modulating of tobacco smoking on CHRNA3. Furthermore, those individuals being passive smokers in childhood (passive smoking from parents) with rs6495309C genotypes would face a more noticeable high risk of lung cancer as well as COPD, reflecting a hazard risk of parents’ smoking on children’s healthy. Previous studies had controversial results of the association between *CHRNA3* SNPs and lung diseases risk in never smokers [16], [17], [25], mainly due to the bias from different passive smoking status. Here, we showed that the association was significant in passive smoker but not in smoke avoider, suggesting that the CHRNA3 SNPs may only exert their genetic effect in smoking-related population.

As we know, COPD and lung cancer are the most striking smoking-related diseases, and COPD is considered to be an important risk factor of lung cancer [8]. Here, this functional causal SNP rs6495309T>C shared by COPD and lung cancer, supported an intrinsic linkage of smoking’s effect on these diseases. Furthermore, COPD cases with rs6495309C genotypes would suffer an intuitively higher risk of lung cancer, indicating a possible predisposition of COPD patients to development of lung cancer in those genotypes carriers. Yet, studies deeply into investigating and verifying those indicated genetic markers to predict the lung cancer risk in COPD patients are essential.

The present study has several strengths. In previous lung cancer case-control studies, the controls were cancer-free subjects without excluding the COPD patients [15], [16], [18], [19], [25], [33], and thus these studies could not avoid the possible confounding bias on evaluating the association between the genetic variants and lung cancer risk. Here, in our designed case-control study, the controls were all cancer-free and with normal pulmonary function, which allowed us to simultaneously compare the COPD and lung cancer groups with the same control group. Such a study design helped us to unravel the intrinsic linkage between COPD and lung cancer. Furthermore, our study had high statistical power for both COPD and lung cancer association analysis (98.3% for lung cancer, 94.5% for COPD). However, there were also some limitations because this was a hospital-based case-control study, there must be selection bias or information bias. In addition, because the controls were age (±5 years) and sex frequency matched with COPD patients, significant deviation in age distribution was observed between lung cancer cases and controls, which may lead to some confounding.

In conclusion, our data revealed a shared susceptible SNP rs6495309T>C and its interaction with smoking or passive smoking in association with the risk and prognosis for both COPD and lung cancer in Chinese populations. The SNP rs6495309T>C, once validated by others would be a useful biomarker to predict the risk and prognosis of COPD and lung cancer.

## Supporting Information

Figure S1 **Haplotype block and linkage disequilibrium (LD) structure for SNPs in Chr15∶76511510-76765500 in CHB (Chinese Han Beijing) population.**
(TIF)

Figure S2 **Haplotype block and linkage disequilibrium (LD) structure for the seven GWAS associated SNP shared by COPD and lung cancer.**
(TIF)

Figure S3 **Genomic structure of** ***CHRNA3*** **, and genotyping of rs6495309T>C, rs1051730G>A by PCR-RFLP as well as direct sequencing.**
(TIF)

Figure S4 **Stratification analysis of the rs6495309T>C polymorphism for COPD or lung cancer risk.** *P* value for the homogeneity test in each stratum was tested by Breslow-Day Test and a multiplicative interaction model was applied for the interaction analysis. The increased risk caused by the rs6495309T>C transition was more pronounced in smokers for both COPD and lung cancer, while the effect of rs6495309T>C genotypes in passive smokers was pronounced for lung cancer risk but not significant for CODP risk.
(TIF)

Figure S5 **Stratification analysis of the rs6495309T>C polymorphism for lung cancer survival.** Cox model was used to calculate the HR of rs6495309T>C genotypes and *P* value of its interaction with the possible enviromental factors for lung cancer survival.
(TIF)

Table S1 **Frequency distributions of selected variables in COPD, lung cancer cases and controls.**
(DOC)

Table S2 **The Effects of patients’ characteristics and clinical features on the annual decline of pre-bronchodilator FEV1.**
(DOC)

Table S3 **Analysis of the Effects of patients’ characteristics and clinical features on lung cancer survival.**
(DOC)

Appendix S1 **Additional details of methods.**
(DOC)

File S1 **The self-made excel file to search the shared loci by COPD and lung cancer based on the data of published GWAS in COPD or lung cancer.**
(XLS)
