# Supplementary figures and images for: Functional Polymorphisms of CHRNA3 Predict Risks of Chronic Obstructive Pulmonary Disease and Lung Cancer in Chinese
Source: PLoS One. 2012 Oct 3;7(10):e46071. doi: 10.1371/journal.pone.0046071 (PMC3463594; doi:10.1371/journal.pone.0046071)

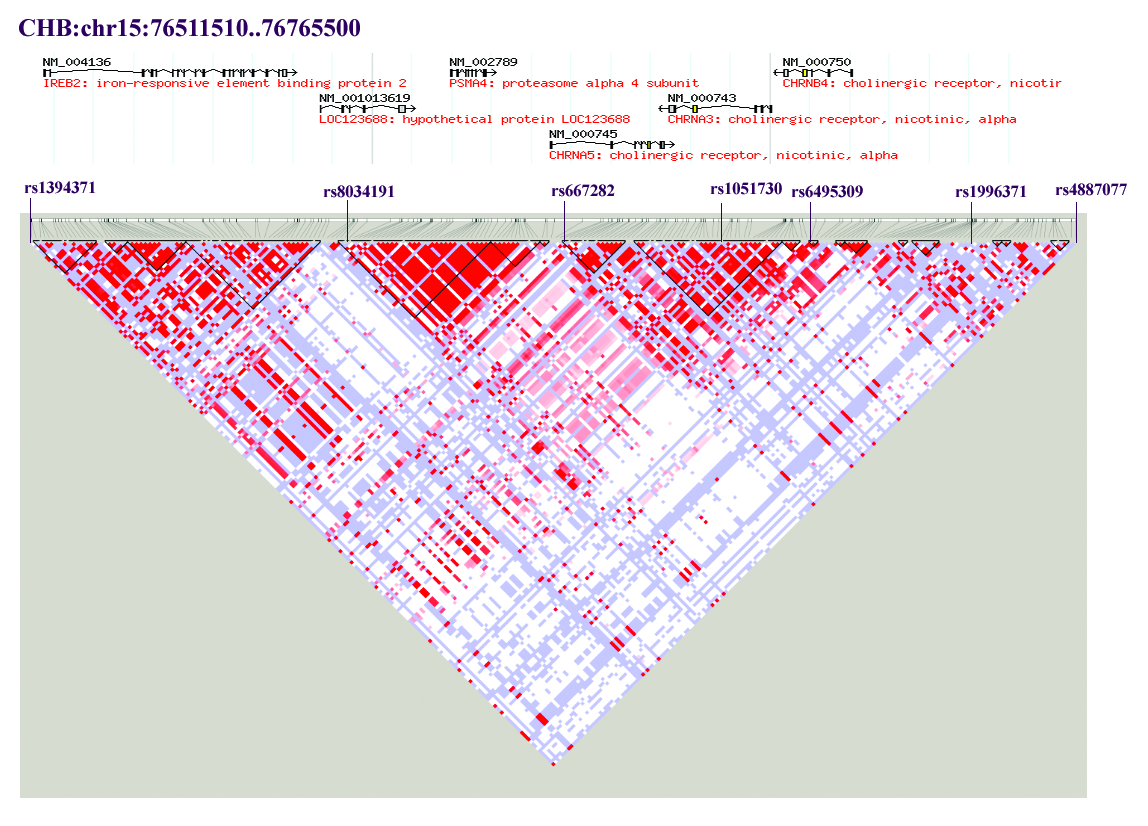

Supplement: Figure S1 — Haplotype block and linkage disequilibrium (LD) structure for SNPs in Chr15∶76511510-76765500 in CHB (Chinese Han Beijing) population. (TIF) [file pone.0046071.s001.tif]

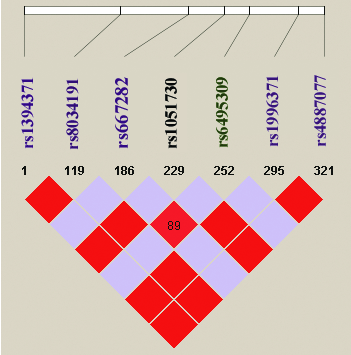

Supplement: Figure S2 — Haplotype block and linkage disequilibrium (LD) structure for the seven GWAS associated SNP shared by COPD and lung cancer. (TIF) [file pone.0046071.s002.tif]

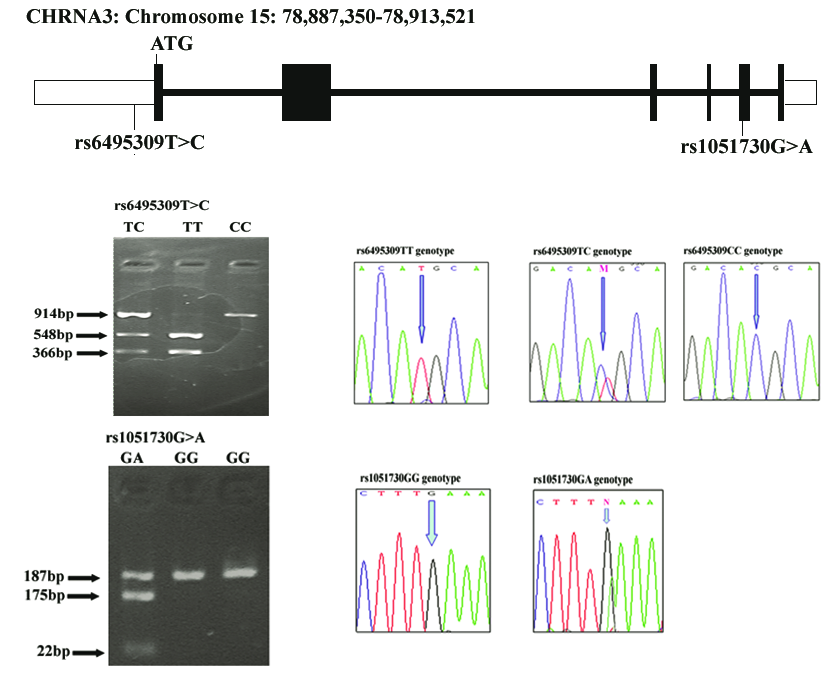

Supplement: Figure S3 — Genomic structure of CHRNA3 , and genotyping of rs6495309T>C, rs1051730G>A by PCR-RFLP as well as direct sequencing. (TIF) [file pone.0046071.s003.tif]

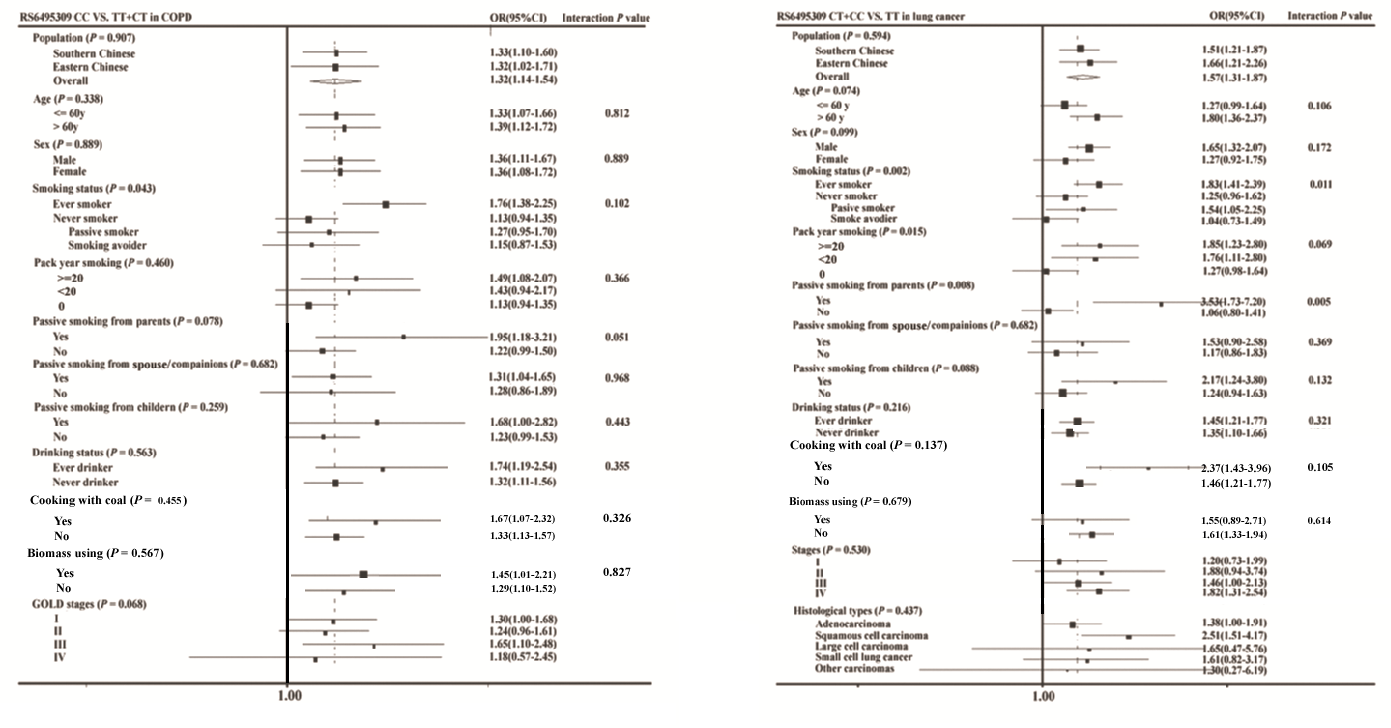

Supplement: Figure S4 — Stratification analysis of the rs6495309T>C polymorphism for COPD or lung cancer risk. P value for the homogeneity test in each stratum was tested by Breslow-Day Test and a multiplicative interaction model was applied for the interaction analysis. The increased risk caused by the rs6495309T>C transition was more pronounced in smokers for both COPD and lung cancer, while the effect of rs6495309T>C genotypes in passive smokers was pronounced for lung cancer risk but not significant for CODP risk. (TIF) [file pone.0046071.s004.tif]

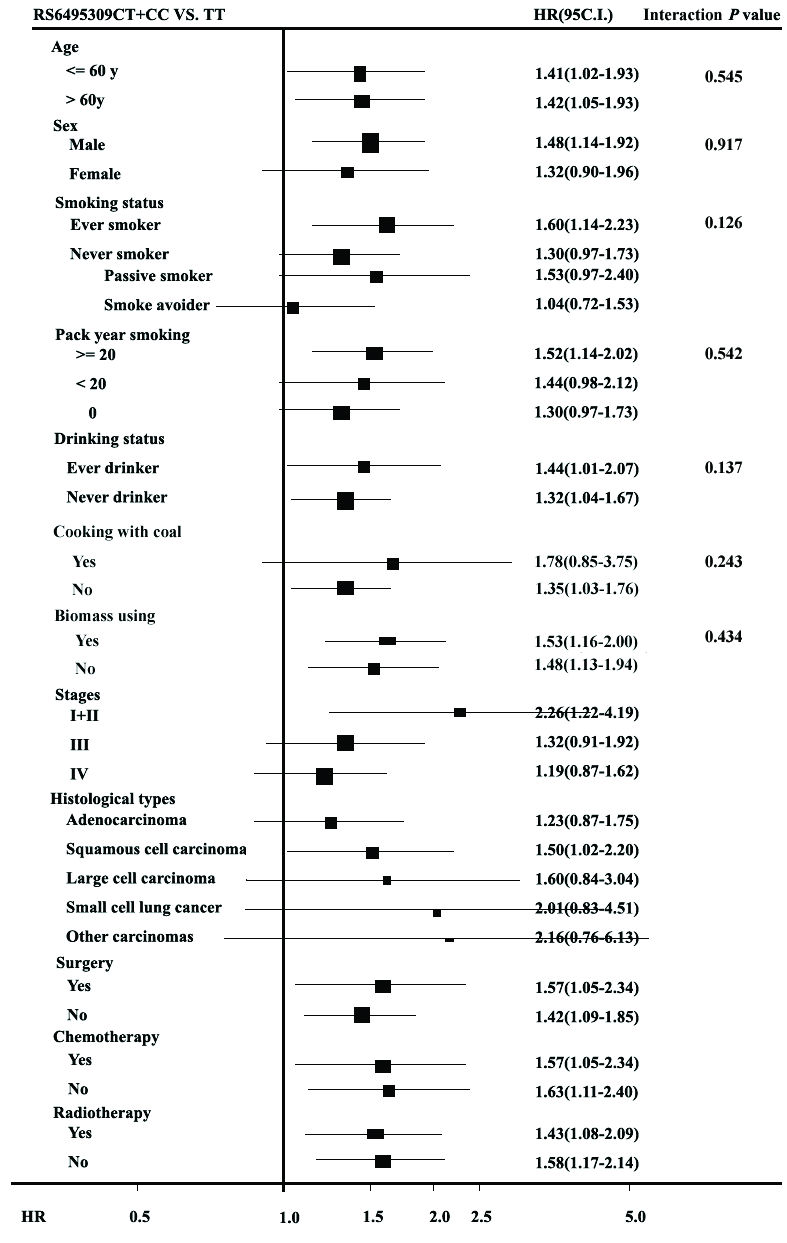

Supplement: Figure S5 — Stratification analysis of the rs6495309T>C polymorphism for lung cancer survival. Cox model was used to calculate the HR of rs6495309T>C genotypes and P value of its interaction with the possible enviromental factors for lung cancer survival. (TIF) [file pone.0046071.s005.tif]
