# Supplementary material for: Functional Polymorphisms of CHRNA3 Predict Risks of Chronic Obstructive Pulmonary Disease and Lung Cancer in Chinese
Source: PLoS One. 2012 Oct 3;7(10):e46071. doi: 10.1371/journal.pone.0046071 (PMC3463594; doi:10.1371/journal.pone.0046071)
Supplement: Table S1 — Frequency distributions of selected variables in COPD, lung cancer cases and controls. (DOC) [file pone.0046071.s006.doc]

**Table S1. Frequency distributions of selected variables in COPD, lung cancer cases and controls**

|  | **Discovery Set (Southern Chinese)** | | | | | | |  | | **Validation Set (Eastern Chinese)** | | | | | |
| --- | --- | --- | --- | --- | --- | --- | --- | --- | --- | --- | --- | --- | --- | --- | --- |
| Variables | Controls  n = 1061  n (%) | COPD  n = 1025  n (%) | *P ^a^* |  | Lung cancer  n = 1056  n (%) | *P ^b^* |  | | Controls  n = 616  n (%) | | COPD  n = 486  n (%) | *P ^a^* |  | Lung cancer  n = 503  n (%) | *P ^b^* |
| Age (years) |  |  |  |  |  |  |  | |  | |  |  |  |  |  |
| ≤ 60 | 507(47.8) | 463(45.2) | 0.231 |  | 536(50.8) | 0.171 |  | | 292(47.4) | | 241(49.6) | 0.471 |  | 273(54.3) | 0.022 |
| > 60 | 554(52.2) | 562(54.8) |  |  | 520(49.2) |  |  | | 324(52.6) | | 245(50.4) |  |  | 230(45.7) |  |
| Sex |  |  |  |  |  |  |  | |  | |  |  |  |  |  |
| Male | 638(60.1) | 610(59.5) | 0.773 |  | 746(70.6) | <0.001 |  | | 345(56.0) | | 273(56.2) | 0.956 |  | 345(66.6) | <0.001 |
| Female | 423(39.9) | 415(40.5) |  |  | 310(29.4) |  |  | | 271(44.0) | | 213(43.8) |  |  | 158(31.4) |  |
| Smoking status |  |  |  |  |  |  |  | |  | |  |  |  |  |  |
| Ever smoker | 424(40.0) | 499(48.7) | <0.001*^c^* |  | 601(56.9) | <0.001 *^c^* |  | | 227(36.8) | | 215(44.2) | 0.013 *^c^* |  | 223(44.4) | 0.011 *^c^* |
| Current smoker | 245(23.1) | 283(27.6) |  |  | 394(37.3) |  |  | | 132(21.4) | | 145(29.8) |  |  | 118(23.5) |  |
| Former smoker | 179(16.9) | 216(21.1) |  |  | 207(19.6) |  |  | | 95(15.4) | | 70(14.4) |  |  | 105(20.9) |  |
| Never smoker | 637(60.0) | 526(51.3) |  |  | 455(43.1) |  |  | | 389(63.2) | | 271(55.8) |  |  | 280(55.6) |  |
| Passive smokers | 283(44.4) | 245(46.6) | 0.463 *^d^* |  | 227(49.9) | 0.074 *^d^* |  | | 186(47.8) | | 152(56.1) | 0.036 *^d^* |  | 144(51.4) | 0.356 *^d^* |
| Smoke avoider | 354(55.6) | 281(53.4) |  |  | 228(50.1) |  |  | | 203(52.2) | | 119(43.9) |  |  | 136(48.6) |  |
| Pack-year smoked |  |  |  |  |  |  |  | |  | |  |  |  |  |  |
| >=20 | 214(20.2) | 314(30.6) | <0.001 |  | 425(40.2) | <0.001 |  | | 129(20.9) | | 148(30.5) | 0.025 |  | 165(32.8) | 0.002 |
| <20 | 210(19.8) | 185(18.1) |  |  | 176(16.7) |  |  | | 98(15.9) | | 67(15.2) |  |  | 58 (11.5) |  |
| 0 | 637(60.0) | 526(51.3) |  |  | 455(43.1) |  |  | | 389(63.2) | | 271(55.8) |  |  | 280(55.7) |  |
| Passive smoking source |  |  |  |  |  |  |  | |  | |  |  |  |  |  |
| parents |  |  |  |  |  |  |  | |  | |  |  |  |  |  |
| Yes | 46(16.2) | 57(23.3) | 0.043 |  | 33(14.5) | 0.594 |  | | 23(12.4) | | 37(24.3) | 0.004 |  | 22(15.3) | 0.445 |
| No | 237(83.8) | 188(76.7) |  |  | 194(85.5) |  |  | | 163(87.6) | | 115(75.7) |  |  | 122(84.7) |  |
| spouse/colleagues |  |  |  |  |  |  |  | |  | |  |  |  |  |  |
| Yes | 146(51.6) | 144(58.8) | 0.098 |  | 133(58.6) | 0.115 |  | | 92(49.5) | | 95(62.5) | 0.017 |  | 110(76.4) | <0.001 |
| No | 137(48.4) | 101(41.2) |  |  | 94(41.4) |  |  | | 94(50.5) | | 57(37.5) |  |  | 34(23.6) |  |
| children |  |  |  |  |  |  |  | |  | |  |  |  |  |  |
| Yes | 127(44.9) | 89(36.3) | 0.046 |  | 97(42.7) | 0.628 |  | | 81(43.6) | | 58(38.2) | 0.316 |  | 31(21.5) | <0.001 |
| No | 156(55.1) | 156(63.7) |  |  | 130(57.3) |  |  | | 105(56.4) | | 94(61.8) |  |  | 113(78.5) |  |
| Drinking status |  |  |  |  |  |  |  | |  | |  |  |  |  |  |
| Ever | 209(19.7) | 186(18.1) | 0.066 |  | 229(21.7) | 0.259 |  | | 127(20.6) | | 97(20.0) | 0.788 |  | 64(12.7) | <0.001 |
| Never | 852(80.3) | 839(81.9) |  |  | 827(78.3) |  |  | | 489(79.4) | | 389(80.0) |  |  | 439(87.3) |  |
| Cooking with coal |  |  |  |  |  |  |  | |  | |  |  |  |  |  |
| No | 935(88.1) | 861(84.0) | 0.007 |  | 857(81.2) | <0.001 |  | | 546(88.6) | | 412(84.8) | 0.059 |  | 422(83.9) | 0.021 |
| Yes | 126(11.9) | 164(16.0) |  |  | 199(18.8) |  |  | | 70(11.4) | | 74(15.2) |  |  | 81(16.1) |  |
| Biomass using |  |  |  |  |  |  |  | |  | |  |  |  |  |  |
| No | 970(91.4) | 851(83.0) | <0.001 |  | 910(86.2) | <0.001 |  | | 570(92.5) | | 417(85.8) | <0.001 |  | 441(87.7) | 0.006 |
| Yes | 91(8.6) | 174(17.0) |  |  | 146(13.8) |  |  | | 46(7.5) | | 69(14.2) |  |  | 62(12.3) |  |
| Pre-FEV1 (mean±SD, L) | 2.29±0.63 | 1.67±0.62 | <0.001 |  |  |  |  | | 2.17±0.60 | | 1.74±0.58 | <0.001 |  |  |  |
| Post-FEV1 (mean±SD, L) | 2.52±0.52 | 1.80±0.63 | <0.001 |  |  |  |  | | 2.41±0.63 | | 1.93±0.61 | <0.001 |  |  |  |
| Post-FEV1% predicted (mean±SD) | 100.4±17.21 | 48.91±14.78 | <0.001 |  |  |  |  | | 101.3±13.91 | | 52.18±13.18 | <0.001 |  |  |  |
| Post-FEV1/FVC (mean±SD) | 0.81±0.06 | 0.59±0.09 | <0.001 |  |  |  |  | | 0.82±0.06 | | 0.60±0.09 | <0.001 |  |  |  |

*^a^ P* values for a two-sided χ^2^ test or t test between COPD and control.

*^b^* *P* values for a two-sided χ^2^ test or t test between lung cancer and control.

*^c^ P* values for differences of smoking status between smoker and never smoker.

*^d^ P* values for differences of never smoker between passive smoker and smoke avoider.
