# Supplementary material for: Functional Polymorphisms of CHRNA3 Predict Risks of Chronic Obstructive Pulmonary Disease and Lung Cancer in Chinese
Source: PLoS One. 2012 Oct 3;7(10):e46071. doi: 10.1371/journal.pone.0046071 (PMC3463594; doi:10.1371/journal.pone.0046071)
Supplement: Table S2 — The Effects of patients’ characteristics and clinical features on the annual decline of pre-bronchodilator FEV1. (DOC) [file pone.0046071.s007.doc]

**Table S2.** The Effects of patients’ characteristics and clinical features on the annual decline of pre-bronchodilator FEV1

|  | **COPD patients** | | | | **Controls** | | | | |
| --- | --- | --- | --- | --- | --- | --- | --- | --- | --- |
| Variables | n (%) | mean ± SD | β (SE) *^a^* | *P* value |  | n (%) | mean ± SD | β (SE) *^a^* | *P* value |
| Total no. of subjects | 116 |  |  |  |  | 357 |  |  |  |
| Age, years |  |  |  |  |  |  |  |  |  |
| ≤ 60 | 22 (13.2) | 0.088 ± 0.074 | -0.057(0.116) | 0.643 |  | 156 (43.7) | 0.085 ± 0.113 | 0.026(0.058) | 0.629 |
| >60 | 145 (86.8) | 0.107 ± 0.103 |  |  |  | 301 (53.6) | 0.092 ± 0.120 |  |  |
| Sex |  |  |  |  |  |  |  |  |  |
| Male | 83 (71.6) | 0.103 ± 0.094 | 0.004(2.711) | 0.979 |  | 162 (45.4) | 0.104 ± 0.131 | -0.101(1.247) | **0.032** |
| Female | 33 (28.4) | 0.091 ± 0.079 |  |  |  | 195 (54.6) | 0.077 ± 0.062 |  |  |
| Smoking status |  |  |  |  |  |  |  |  |  |
| Never | 40 (34.5) | 0.084 ± 0.085 | 0.125(0.872) | **0.012** |  | 223 (62.5) | 0.079 ± 0.113 | 0.093(1.002) | **0.041** |
| Ever | 76 (65.5) | 0.112 ± 0.093 |  |  |  | 134 (37.5) | 0.105 ± 0.124 |  |  |
| Pack-year smoked |  |  |  |  |  |  |  |  |  |
| 0 | 40 (34.5) | 0.084 ± 0.085 | 0.138(0.044) | **0.043** |  | 230 (64.4) | 0.084 ± 0.112 | 0.136(1.702) | **0.049** |
| <20 | 24 (15.5) | 0.093 ± 0.078 |  |  |  | 71 (19.9) | 0.091 ± 0.125 |  |  |
| ≥20 | 52 (44.8) | 0.113 ± 0.100 |  |  |  | 56 (15.7) | 0.109 ± 0.131 |  |  |
| Passive smoking |  |  |  |  |  |  |  |  |  |
| No | 16 (40.0) | 0.092 ± 0.106 | 0.015(0.035) | 0.944 |  | 106 (46.2) | 0.089 ± 0.114 | -0.034(0.015) | 0.614 |
| Yes | 24 (60.0) | 0.094 ± 0.072 |  |  |  | 124 (53.8) | 0.081 ± 0.113 |  |  |
| Drinking status |  |  |  |  |  |  |  |  |  |
| Never | 76 (65.5) | 0.092 ± 0.077 | 0.119(1.426) | 0.382 |  | 286 (80.1) | 0.089 ± 0.120 | -0.030(1.677) | 0.596 |
| Ever | 40 (34.5) | 0.115 ± 0.111 |  |  |  | 71 (19.9) | 0.090 ± 0.108 |  |  |
| Cooking with coal |  |  |  |  |  |  |  |  |  |
| No | 103(88.8) | 0.074 ± 0.064 | 0.023(0.035) | 0.520 |  | 320(89.6) | 0.076 ± 0.098 | -0.012(0.022) | 0.575 |
| Yes | 13(11.2) | 0.102 ± 0.092 |  |  |  | 37(11.4) | 0.091 ± 0.119 |  |  |
| Biomass using |  |  |  |  |  |  |  |  |  |
| No | 105(90.5) | 0.081 ± 0.044 | 0.153(0.065) | **0.020** |  | 331(92.7) | 0.083 ± 0.101 | 0.031(0.019) | 0.098 |
| Yes | 11(9.5) | 0.112 ± 0.065 |  |  |  | 26(7.3) | 0.099 ± 0.080 |  |  |
| Gold Stages |  |  |  |  |  |  |  |  |  |
| I | 41 (24.6) | 0.085 ± 0.056 | 0.268(0.013) | **0.033** |  |  |  |  |  |
| II | 78 (46.7) | 0.099 ± 0.092 |  |  |  |  |  |  |  |
| III | 37 (22.1) | 0.114 ± 0.107 |  |  |  |  |  |  |  |
| IV | 11 (6.6) | 0.118 ± 0.215 |  |  |  |  |  |  |  |

*^a^* Linear regress analysis with adjustment for age, sex, smoke status, drinking status.
