# Supplementary material for: Functional Polymorphisms of CHRNA3 Predict Risks of Chronic Obstructive Pulmonary Disease and Lung Cancer in Chinese
Source: PLoS One. 2012 Oct 3;7(10):e46071. doi: 10.1371/journal.pone.0046071 (PMC3463594; doi:10.1371/journal.pone.0046071)
Supplement: Table S3 — Analysis of the Effects of patients’ characteristics and clinical features on lung cancer survival. (DOC) [file pone.0046071.s008.doc]

**Table S3.** Analysis of the Effects of patients’ characteristics and clinical features on lung cancer survival

| Variables |  | Discovery set  (Southern Chinese) | |  | Validation set  (Eastern Chinese) | |  |  | MST  (months) | Log-rank  *P* value | Cox model  HR (95%CI)*^a^* |
| --- | --- | --- | --- | --- | --- | --- | --- | --- | --- | --- | --- |
|  |  | cases  n (%) | No. of  death |  | cases  n (%) | No. of  death |  |  |
| Total |  | 510 | 413 |  | 296 | 203 |  |  |  |  |  |
| Age (years) |  |  |  |  |  |  |  |  |  | **0.029** |  |
| ≤ 60 |  | 229 (44.9) | 190 |  | 151 (51.0) | 92 |  |  | 15 |  | 1.00 (ref.) |
| > 60 |  | 281 (50.1) | 223 |  | 145 (49.0) | 111 |  |  | 11 |  | **1.16 (0.99-1.37)** |
| Sex |  |  |  |  |  |  |  |  |  | 0.076 |  |
| Male |  | 366 (71.8) | 301 |  | 200 (67.6) | 138 |  |  | 12 |  | 1.00 (ref.) |
| Female |  | 144 (28.2) | 112 |  | 96 (32.4) | 65 |  |  | 15 |  | 0.88 (0.71-1.07) |
| Family history of cancer |  |  |  |  |  |  |  |  |  |  |  |
| No |  | 461 (90.4) | 372 |  | 285 (96.3) | 197 |  |  | 13 | 0.271 | 1.00 (ref.) |
| Yes |  | 49 (9.6) | 41 |  | 11 (3.7) | 6 |  |  | 16 |  | 0.82 (0.61-1.11) |
| Family history of lung cancer |  |  |  |  |  |  |  |  |  |  |  |
| No |  | 489 (95.9) | 397 |  | 292 (98.6) | 201 |  |  | 13 | 0.154 | 1.00 (ref.) |
| Yes |  | 21 (4.1) | 16 |  | 4 (1.4) | 2 |  |  | 17 |  | 0.67 (0.42-1.07) |
| Smoking status |  |  |  |  |  |  |  |  |  | **0.001** |  |
| Never |  | 211 (41.4) | 167 |  | 165 (55.7) | 106 |  |  | 15 |  | 1.00 (ref.) |
| Ever |  | 299 (58.6) | 246 |  | 131 (42.3) | 97 |  |  | 11 |  | **1.28 (1.08-1.50)** |
| Pack-years smoked |  |  |  |  |  |  |  |  |  | **0.018** |  |
| 0 |  | 221 (43.3) | 167 |  | 166 (56.1) | 107 |  |  | 15 |  | 1.00 (ref.) |
| <20 |  | 59 (11.6) | 43 |  | 24 (8.1) | 17 |  |  | 13 |  | 1.15 (0.89-1.51) |
| ≥20 |  | 240 (47.1) | 203 |  | 106 (35.8) | 79 |  |  | 11 |  | **1.14 (1.05-1.24)** |
| Passive smoking |  |  |  |  |  |  |  |  |  | 0.493 |  |
| No |  | 97 (37.2) | 76 |  | 73 (44.2) | 40 |  |  | 16 |  | 1.00 (ref.) |
| Yes |  | 164 (62.8) | 136 |  | 92 (55.8) | 67 |  |  | 14 |  | 1.04 (0.82-1.32) |
| Drinking status |  |  |  |  |  |  |  |  |  | 0.240 |  |
| Never |  | 401 (78.6) | 322 |  | 262 (88.5) | 178 |  |  | 13 |  | 1.00 (ref.) |
| Ever |  | 109 (21.4) | 91 |  | 34 (11.5) | 25 |  |  | 11 |  | 1.01 (0.81-1.25) |
| Cooking with coal |  |  |  |  |  |  |  |  |  |  |  |
| No |  | 436(85.5) | 352 |  | 250(84.5) | 173 |  |  | 12 | 0.232 | 1.00 (ref.) |
| Yes |  | 74(14.5) | 61 |  | 46(15.5) | 30 |  |  | 9 |  | 1.07(0.72-1.58) |
| Biomass using |  |  |  |  |  |  |  |  |  |  |  |
| No |  | 455(89.2) | 362 |  | 265(89.5) | 178 |  |  | 11 |  | 1.00 (ref.) |
| Yes |  | 55(10.8) | 51 |  | 31(10.5) | 25 |  |  | 8 |  | 1.10(0.84-1.45) |
| Surgery |  |  |  |  |  |  |  |  |  | **<0.001** |  |
| No |  | 334 (65.5) | 289 |  | 162 (54.7) | 133 |  |  | 11 |  | 1.00 (ref.) |
| Yes |  | 176 (34.5) | 124 |  | 134 (45.3) | 70 |  |  | 20 |  | **0.59(0.50-0.70)** |
| Chemotherapy |  |  |  |  |  |  |  |  |  | **0.017** |  |
| No |  | 203 (39.8) | 162 |  | 85 (28.7) | 60 |  |  | 9 |  | 1.00 (ref.) |
| Yes |  | 307 (60.2) | 251 |  | 211 (71.3) | 143 |  |  | 15 |  | **0.84(0.71-0.99)** |
| Radiotherapy |  |  |  |  |  |  |  |  |  | **0.011** |  |
| No |  | 274 (53.7) | 218 |  | 118 (39.9) | 82 |  |  | 10 |  | 1.00 (ref.) |
| Yes |  | 236 (46.3) | 195 |  | 178 (60.1) | 121 |  |  | 15 |  | **0.85(0.72-0.99)** |
| Stages |  |  |  |  |  |  |  |  |  | **<0.001** |  |
| I+ II |  | 96 (18.8) | 56 |  | 58 (19.6) | 33 |  |  | 22 |  | 1.00 (ref.) |
| III |  | 169 (33.1) | 145 |  | 94 (31.8) | 63 |  |  | 12 |  | **1.71 (1.33-2.20)** |
| IV |  | 245 (48.1) | 212 |  | 144 (48.6) | 107 |  |  | 11 |  | **1.90 (1.50-2.41)** |
| Histological types |  |  |  |  |  |  |  |  |  | 0.588 |  |
| Adenocarcinoma |  | 179 (35.1) | 144 |  | 132 (44.6) | 89 |  |  | 12 |  |  |
| Squamous cell carcinoma |  | 164 (32.2) | 133 |  | 95 (32.1) | 65 |  |  | 12 |  |  |
| Large cell carcinoma |  | 24 (4.7) | 17 |  | 17 (5.7) | 12 |  |  | 14 |  |  |
| Small cell lung cancer |  | 62 (12.1) | 52 |  | 39 (13.2) | 30 |  |  | 10 |  |  |
| Other carcinomas *^b^* |  | 81 (15.9) | 67 |  | 13 (4.4) | 7 |  |  | 3 |  |  |

Abbrevations: MST, median survival time; HR, hazard ratio;

*^a^* Adjusted in a univariate Cox regression analysis that included age, sex, smoke, drink, histology, stage, surgery, chemotherapy, and radiotherapy status.

*^b^* Mixed-cell or undifferentiated carcinoma.
