# Supplementary material for: Functional Polymorphisms of CHRNA3 Predict Risks of Chronic Obstructive Pulmonary Disease and Lung Cancer in Chinese
Source: PLoS One. 2012 Oct 3;7(10):e46071. doi: 10.1371/journal.pone.0046071 (PMC3463594; doi:10.1371/journal.pone.0046071)
Supplement: Appendix S1 — Additional details of methods. (DOC) [file pone.0046071.s009.doc]

**Appendix S1: additional details of methods**

**1. *Sample collection***

A**ll COPD subjects were diagnosed based on medical history, chest radiographic findings, physical examination and spirometric data. Inclusion criteria for COPD included the following: chronic airway symptoms and signs, such as chronic cough, dyspnea, sputum production, wheezing, and chronic airway obstruction, defined as a FEV1/forced vital capacity (FVC) of <70% after inhalation of** 400μg **salbutamol.** In the discovery set, 697 cases of southern Chinese were recruited from three communities (Liwan, Xicun and Zhanqian communities) in Guangzhou based on the cross-sectional surveies of COPD between February 2002 and June 2008 with a response rate of about 81%, 328 cases were recruited from municipal hospitals in Guangzhou (the Guangzhou Chest Hospital, The third Affiliated Hospital of Guangzhou Medical University, the third Affiliated Hospital of Sun Yat-sen University) between December 2007 and June 2010 with 91% response rate. And there were 359 (35.0%) cases of stage I, 356 (34.7%) stage II, 217 (21.2%) stage III, and 93 (9.1%) stage IV. The normal controls (**FEV1/ FVC > 70%**) were randomly picked from 6000 individuals participating in the community cross-sectional survey of COPD. In the validation set, the 486 cases of COPD in eastern Chinese were recruited the Second Affiliate Hospital of Soochow University (Suzhou) with a response rate of 83% during the July 2009 to June 2011 and there were 164 (33.8%) cases of stage I, 157 (32.4%) stage II, 112 (23.1%) stage III, and 52 (10.7%) stage IV. The 616 normal pulmonary function controls were randomly selected from a database consisting of 3,500 individuals based on a physical examination with an 81% response rate.

**2. *Definition of pre-existing COPD and smoking status***

Lung cancer patients with physician-diagnosed COPD at least one year before lung cancer diagnosis were defined to have been pre-existing COPD, among them, 148 and 69 lung cancer patients from the discovery and validation set had been pre-existing COPD; Those subjects who had smoked <100 cigarettes in their lifetime were defined as never smokers; others were smokers and those smokers who had quit for >1 year before enrollment were considered former smokers, and the remaining smokers were defined as current smokers. For smokers, a questionnaire was administered to ask about the pack of cigarettes per year. Similarly, participants who had consumed alcoholic beverages at least once a week for ≥1 year previously were defined as drinkers, and the else as never drinkers. A passive smoker was defined as those never smokes who inhaled tobacco smog in ambient environment more than 1 hour/day in one day a week or more according to WHO Specification Guidelines, and the remaining never smokers were defined as smoke avoiders[^1^](#_ENREF_1). The source of passive smoking was further categorized as (i) from parents in their childhood; (ii) from spouse or colleagues; (iii) from his or her children in their elderly age.

**3.** ***Spirometry performing***

Spirometry was performed by trained technicians with a portable spirometer (Micro Medical, Chatham, Kent) according to the criteria recommended by the American Thoracic Society [^2^](#_ENREF_2) and the Europe Respiratory Society [^3^](#_ENREF_3). The largest pre-bronchodilator FEV1 and FVC were chosen from at least three acceptable and two reproducible measurements, which met the criteria for each individual. The post-bronchodilator spirometry was obtained after inhalation of 400μg of salbutamol for 20 minutes (Ventolin, GlaxoSmithKline) via a 500 ml spacer for those participants whose pre-FEV1/FVC was <70%. Furthermore, the predicted values of FEV1 were estimated as described previously [^4^](#_ENREF_4).

**REFERENCE**

1. Ding D, Wahlgren DR, Liles S, Jones JA, Hughes SC, Hovell MF. Secondhand smoke avoidance by preteens living with smokers: to leave or stay? Addict Behav 2010;35:989-94.

2. Standardization of Spirometry, 1994 Update. American Thoracic Society. Am J Respir Crit Care Med 1995;152:1107-36.

3. Pellegrino R, Viegi G, Brusasco V, Crapo RO, Burgos F, Casaburi R, Coates A, van der Grinten CP, Gustafsson P, Hankinson J, Jensen R, Johnson DC, et al. Interpretative strategies for lung function tests. Eur Respir J 2005;26:948-68.

4. Zheng J, Zhong N. Normative values of pulmonary function testing in Chinese adults. Chin Med J (Engl) 2002;115:50-4.
